# Mapping the habitat refugia of *Isidella elongata* under climate change and trawling impacts to preserve Vulnerable Marine Ecosystems in the Mediterranean

**DOI:** 10.1038/s41598-024-56338-1

**Published:** 2024-03-15

**Authors:** Vincent Georges, Sandrine Vaz, Pierluigi Carbonara, Marie-Claire Fabri, Emanuela Fanelli, Maria Cristina Follesa, Germana Garofalo, Vasilis Gerovasileiou, Angélique Jadaud, Porzia Maiorano, Pilar Marin, Chryssi Mytilineou, Covadonga Orejas, Maria Del Mar Otero, Chris J. Smith, Ioannis Thasitis, Valentina Lauria

**Affiliations:** 1 Institute for Biological Resources and Marine Biotechnologies, CNR IRBIM, Mazara del Vallo, Italy; 2 MARBEC, Univ Montpellier, CNRS, Sète, Ifremer, IRD France; 3 Fondazione COISPA ETS, Via Dei Trulli 18/20, Bari, Italy; 4 Ifremer, Centre de Méditerranée, Département Océanographie Et Dynamique Des Ecosystèmes, 83500 La Seyne Sur Mer, France; 5 Department of Life and Environmental Sciences - DiSVA, Università Politecnica Delle Marche, Ancona, Italy; 6 Dipartimento Scienze, Vita E Ambiente, Università Di Cagliari, Cagliari, Italy; 7 Institute for Environmental Protection and Research (ISPRA), BIO-CIT, Lungomare Cristoforo Colombo N. 4521 (Ex Complesso Roosevelt) Località Addaura, 90149 Palermo, Italy; 8 Department of Environment, Faculty of Environment, Ionian University, 29100 Zakynthos, Greece; 9 Institute of Marine Biological Resources and Inland Waters, Hellenic Centre for Marine Research, Gournes, Greece; 10 Department of Bioscience, Biotechnology and Environnement (DBBA), University of Bari Aldo Moro, Bari, Italy; 11 Independent Expert, Madrid, Spain; 12 Instituto Español de Oceanografia, IEO, Centro Oceanográfico de Gijón, Gijón, Spain; 13 ETC-UMA (University of Malaga), Malaga, Spain; 14 Department of Fisheries and Marine Research, Ministry of Agriculture, Rural Development and Environment, 101 Vithleem Street, 2033 Nicosia, Cyprus; 15 National Biodiversity Future Center, NBFC, Palermo, Italy

**Keywords:** Biogeography, Climate-change ecology, Conservation biology, Ecological modelling, Marine biology

## Abstract

The bamboo-coral *Isidella elongata* is a key habitat-forming species in the deep Mediterranean Sea. This alcyonacean is listed as an indicator of Vulnerable Marine Ecosystems (VMEs) and as Critically Endangered due to bottom trawling impacts. In this work, a modeling approach was used to predict and map the habitat suitability of *I. elongata* in the Mediterranean Sea under current environmental conditions. Occurrence data were modeled as a function of environmental parameters. Using climate change scenarios and fishing effort data, the risk of climate change and fisheries impacts on habitat suitability were estimated, and *climate refugia* were identified. A drastic loss of habitat is predicted, and climate change scenarios suggest a loss of 60% of suitable habitats by 2100. In the central Mediterranean, *climate refugia* overlapped with active fishing grounds. This study represents the first attempt to identify hot spots for the protection of soft bottom Vulnerable Marine Ecosystems for the entire Mediterranean Sea, and highlights areas most at risk from trawling. This work is relevant to the objectives of the EU Marine Strategy Framework and Maritime Spatial Planning Directives, the Biodiversity Strategy for 2030 regarding priority areas for conservation.

## Introduction

The International Guidelines for the Management of Deep-sea Fisheries in the High Seas define Vulnerable Marine Ecosystems (VMEs) as groups of species, communities, or habitats characterized by low resilience, for which short-term or chronic disturbances imply a slow and uncertain recovery^1^. Vulnerable Marine Ecosystems are identified by indicator species, characterized by long-life spans, slow growth rates, long reproductive cycles, and low recruitment^2^. As advised in the EU Marine Strategy Framework Directive and the proposed EU Nature Restoration law^3,4^ the necessary steps to protect VMEs include understanding the role of the environmental factors that shape their distribution. Such knowledge can support adequate marine planning and fisheries policy. Despite some studies have tried to understand the role of the environment and/or fishing impact on the habitat selection of VMEs indicator species at regional scale^5–12^ still their large scale distribution and vulnerability to climate change should be investigated at basin scale, as attempted by Morato et al. 2020^13^ for the North Atlantic Ocean, with considerable habitat loss by 2100 foreseen for several deep-sea coral species under high emission scenarios.

The bamboo coral *Isidella elongata* (Esper, 1788) is a deep-sea alcyonacean associated with bathyal mud biocoenosis (sensu Peres and Picard, 1964^14^), generally found at depths greater than 200 m^15^ Species of this genus have a low growth rate (several millimeters per year) with high longevity (145 ± 10 years)^16–18^ and form considerable aggregations of colonies known as coral gardens or marine animal forests^1,15,19–21^. This species has been acknowledged as a representative taxon indicator of VMEs (as defined in Appendix 17 of the report of the forty-second session of the FAO-GFCM; Resolution GFCM/43/2019/6)^22^ and as a Marine Habitat Type for the Selection of Sites to be included in the National Inventories of Natural Sites of Conservation Interest in the Mediterranean^23^ This species is quasi-endemic in the Mediterranean while occurrences and partially high densities have been detected in the Gulf of Cadiz^24^ It plays a key ecological role as it provides a three-dimensional habitat on compact mud-habitats and supports highly diverse macrofaunal communities^5,9,25–28^ Its presence can influence the availability of resources and biodiversity^5,10,27,29–31^, while providing shelter from predators to fish and crustacean species^27^ that can find a high density of prey within its canopy^30^.

Over the last decades, bottom fisheries have been impacting deep-sea habitats at an increasing pace^32–35^, leading to the decline of deep-sea coral abundance^35–37^. Because of its life-history traits and the co-occurrence with fishery target species, such as the deep-sea shrimps *Aristeus antennatus* and *Aristaeomorpha foliacea*^7,27,38^, this species has been classified as «Critically Endangered» by the IUCN with a decline over 80% of its abundance over the last forty years^39^. A direct effect of demersal fisheries is the mechanical destruction of colonies^40^ Visual surveys have shown how bottom trawling can drastically affect *I. elongata* colonies, while in non-trawled areas, very old and large colonies are present^27^ In the Mediterranean Sea, bottom trawling is forbidden below 1000 m^﻿1^ while completely in some Fisheries Restricted Areas (FRAs) and Marine Protected Areas (MPAs)^41^ However, most of them are coastal, which implies that some of the remaining areas where *I. elongata* is present may be jeopardized. In this context, there is a high risk that this species will disappear from the Mediterranean at shallower depths than 1000 m in the next decades.

Deep-sea corals are also known to be vulnerable to anthropogenic climate change^42,43^, and a considerable habitat loss by 2100 has been foreseen in the North Atlantic^13^. In the Mediterranean Sea, *I. elongata* is associated with relatively stable environmental conditions. However, the potential effect of global warming on habitat loss may happen due to sharper temperature increase, change in circulation patterns and acidification effect^13,44–47^, changing organic matter input to the seafloor, decreasing feeding efficiency, and biocalcification process. More frequent extreme events add physical stress on continental margins^48^, and variation in carbonate compensation depth from acidification may impact calcifying organisms^48^. Consequently, long-term management plans for the protection of *I. elongata* should also consider the potential impact of climate change^37^.

Species Distribution Models (SDMs) have been largely used by conservation organizations and researchers to identify by modeling the suitable habitat where VMEs could occur at regional and global scales^13,49–52^. The main advantage of using such models is the ability to predict the distribution of species over wide geographic regions and project changes under future climate scenarios^53–55^, providing distribution maps that can support management actions by policymakers (e.g., planning of fishing restricted areas^11,56–61^.

The aims of this study are: (1) mapping the current distribution and predict the suitable habitat of *I. elongata* at the Mediterranean scale under present and future conditions; (2) identifying climate refugia (areas of preserved habitat suitability) and estimate their risk from impact of bottom trawling. Such knowledge should support future spatial management plans and measures for VMEs with *I. elongata* in the Mediterranean Sea, either at national or regional levels.

## Material and methods

### Study area

**Figure 1:**
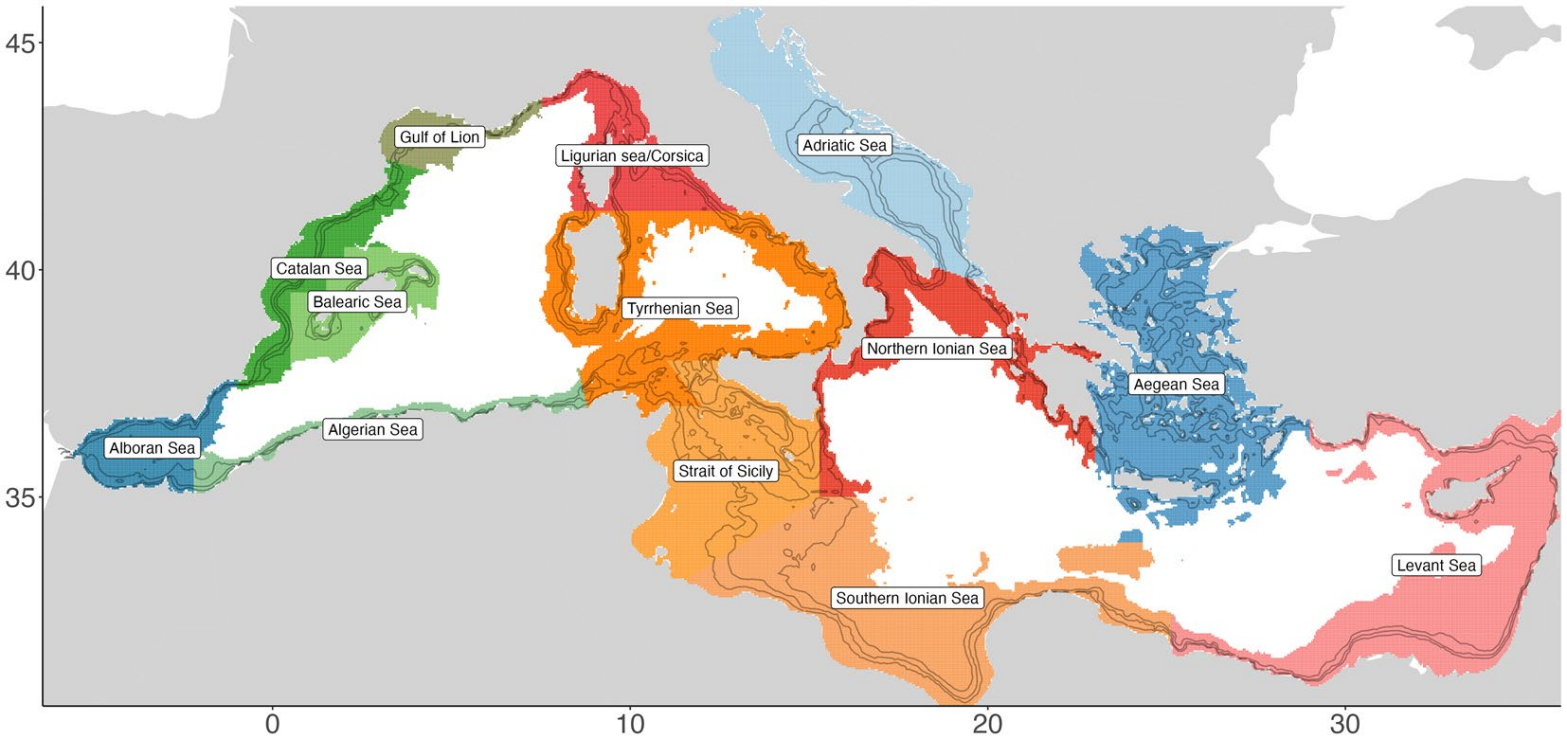
Extent of the study area, with delimitation and terminology of the regional seas. Created using ggplot2 version 3.4.4 https://cran.r-project.org/web/packages/ggplot2/index.html within the R environnement (R Core Team^100^).

The study area covers the Mediterranean Sea (6.5°W—38.0°E; 30.5—45.25°N) constrained to depths less than 2000 m as Fig. 1 shows where also the delimitation of the regional seas are indicated. These are delimited by aggregation of Geographical sub-areas (GSA) of the General Fisheries Commission of the Mediterranean Sea (GFCM).

### Isidella elongata occurrence data

**Figure 2:**
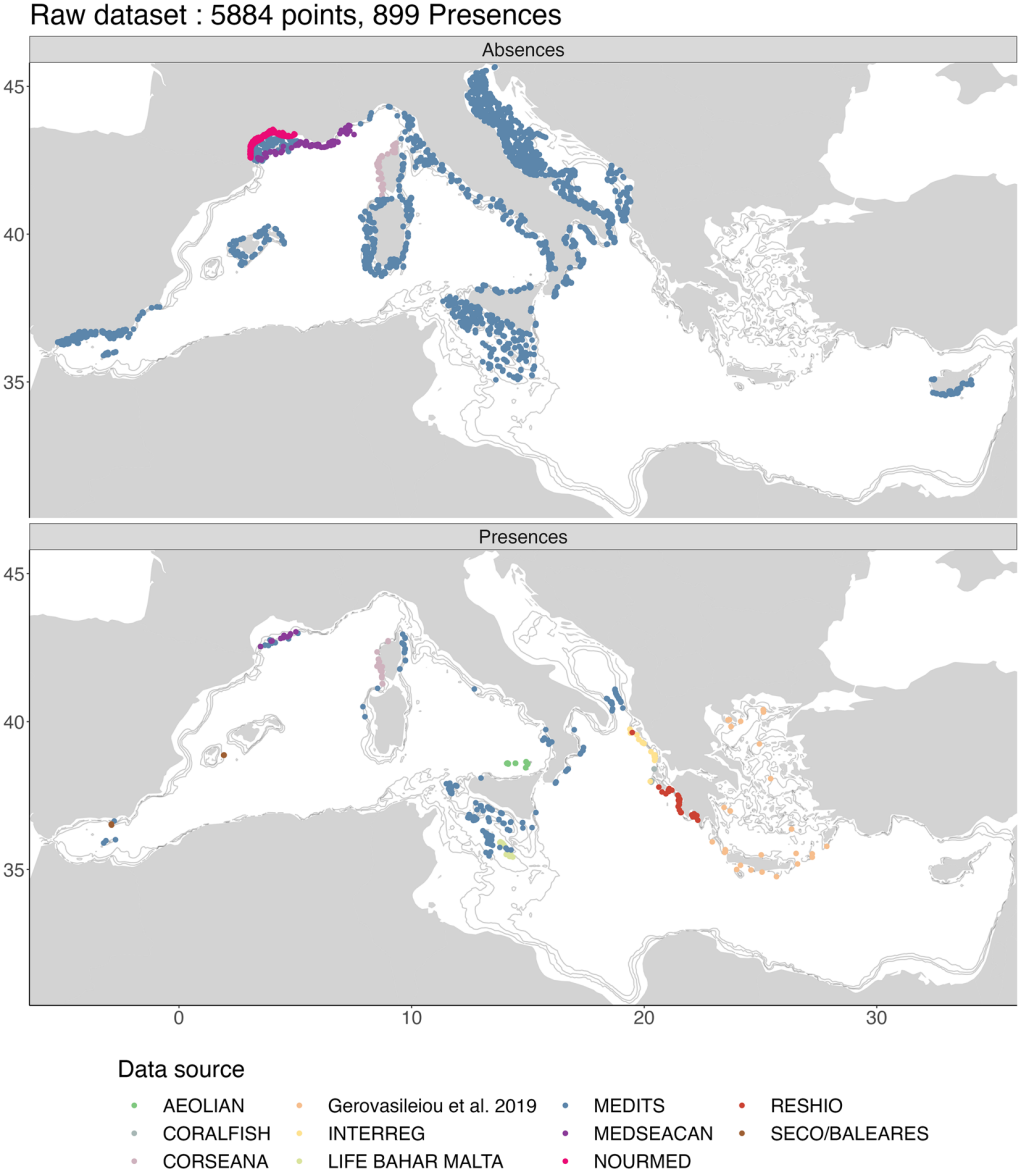
Sampling stations analysed (top map) and stations in which *Isidella elongata* was detected (bottom map) in the Mediterranean Sea, by data source. Created using ggplot2 version 3.4.4 https://cran.r-project.org/web/packages/ggplot2/index.html within the R environnement (R Core Team^100^).

Presence-absence data of *I. elongata* were collated from different sources. A total of 5﻿884 data points including 860 presence records were identified, with data collected between 1891 and 2020, (but the bulk of the data covers the period 2000–2020). The main source of data was the Mediterranean International Trawl Survey program (MEDITS)^62^ a standardized bottom trawling survey carried out in the northern Mediterranean Sea in late spring/early summer since 1994. In addition, other presence/absence records were extracted from the General Fisheries Commission for the Mediterranean Sea (GFCM) database on Sensitive Benthic Habitats and Species that contains presence records of *I. elongata* collected with Remotely Operated Vehicles (ROV) surveys and other fisheries dependent and independent surveys. Other additional points were included from: literature^8^ and Ifremer ROV surveys^40,63^. The spatial distribution of the whole data set is shown in Fig. 2. Details on the source and time-range of data points are shown in supplementary material (Occurrence dataset section).

### Environmental predictors and fishery data

Near-bottom water properties data were extracted from the BIO-ORACLE 2.0 database^64,65^, providing multiple benthic layers on a variety of parameters, as well as projections under climate change scenarios for temperature, salinity and current velocity, all at a native resolution of (0.083 decimal degrees, ~ 8 km at Mediterranean latitudes). Depth was extracted from EMODnet bathymetry data with a resolution of 0.002 degrees (~ 200 m)^66^Slope was derived from the bathymetry layer using the Terrain function of the R raster package^67^ using the method proposed by Horn, 1981 eight neighbors grid calculation^68^ with a resolution of 0.006 degrees (~ 668 m).

The selection of variables used for modeling construction was done based on their ecological relevance to *I. elongata*. Potential collinearity among environmental predictors was investigated using spearman coefficient. All considered layers and eventual reason for being discarded are detailed in the supplementary material (Table S2).

Five environmental predictors were selected for modeling construction, of which three available under future climate change scenario from IPCC RCP 8.5. These were: mean slope, bathymetry, bottom temperature, bottom salinity, and current velocity.

These environmental factors have been shown to be important for the habitat selection of *I. elongata* as well as other deep-sea water corals in the central Mediterranean Sea^7,69^. Bathymetry is a very structuring factor in all marine habitats, representing a proxy for food availability, primary production, and pressure. Slope describes the steepness of the seafloor and is used as a proxy to identify different types of large-scale habitats (such as continental margins canyons or abyssal plains). Low values of slope are associated with flat ocean bottoms (areas of sediment deposition), while higher values indicate potential rocky ledges. Bottom water temperature has been suggested to influence coral calcification rates, physiology and biochemistry, and is the parameters expected to vary the most sharply in the near future^47^, while salinity influences the water column stratification. Water masses together with oceanographic processes, notably internal waves, and current velocity, may be a fundamental driver for the occurrence of benthic suspension feeders, including CWCs^49,70,71^. We consider only the mean, near sea-floor values, for the 2000–2014-time frame in the case of current condition.

Future climate scenario predictors were extracted for temperature, salinity, and current velocity relative to the period 2091–2100, according to the Representative Concentration Pathway 8.5 (RCP 8.5), the most “pessimistic” climate change projection assuming that carbon emissions remain the same with no mitigation, according to the Intergovernmental Panel on Climate Change (IPCC) 2014 report^72^. The maps of the different selected predictors are shown in Fig. S6 in the supplementary material.

A digital continuous map of fishery activity was collected from Automatic Identification System (AIS) signals (per km^2^/year) sent by European bottom trawlers above 15 m in Length Overall (LOA), from Global Fishing Watch v4.^73^. AIS signals provide a good proxy estimation of actual trawling activity, being the best available information to identify fishing grounds on a Mediterranean scale, although information is absent for non-EU-vessels, and the classification of Fishing activity is done algorithmically and thus may contains artifact and imprecisions^50^.

### Data preparation for species distribution modeling

The original presence-absence dataset included a high number of observations (n = 5884). Prior to model construction, data were processed following three steps:

1. Pseudo-absence implementation: while areas of MEDITS coverage provide very valuable survey absences in SDM, some areas contained presence only data due to the sampling effort, (Such as ROV or literature data). To avoid bias in the output, Pseudo absences were implemented at random in these areas and their number was based on the presence/absence ratio of MEDITS data. These areas were: the North-eastern Ionian sea, Aegean sea and Aeolian islands (South Tyrrhenian sea). Details on the pseudo absences implementation are given in the supplementary material. No pseudo-absences were added in areas with survey absences available, and below 1000 m depth in our study area.
2. Data aggregation: we used a resampling grid at the predictors resolution (0.041 decimal degrees, ~ 4 km) to filter the original data, keeping only one randomly chosen data point per grid cell, with priority to presence points when both presence and absence were present in the same cell. This helps mitigating sampling bias and spatial autocorrelation by evening out highly close observations and interannual detection of the same colonies, while removing “noisy absences”, sharing the same conditions as a presence, following the assumption that only one presence of *I. elongata* is enough to consider the environmental conditions suitable. The aggregated dataset contained 2843 points, including 279 presence records.
3. Environmental layers homogenization: these were brought to the same resolution of 0.041 decimal degrees (~ 4 km) to account for geolocation error when extracting environmental conditions to midpoints coordinates from 3 to 5 km trawls from MEDITS data. Using the resample function of the Raster r package, finer scale terrain layers were downscaled using mean values from original scale, and coarser layers were upscaled using bilinear interpolation. This resolution was used for all parameters extraction and subsequent modeling prediction.

### Model development and evaluation

#### Modeling algorithms

Two algorithm were chosen for the model construction Generalized Additive Models (GAMs) using the mgcv R package^75,76^and RandomForest (RFs) using the randomforest R package^77,78^ GAMs are characterized by their use of smooth functions to represent linear and nonlinear relationship between regressors and response variables. Giving greater flexibility over linear models. These were fitted with a binomial family and logit link function with a maximum smoothing degree of 4 (k = 4). Random Forest is a modeling technique based on Classification and Regression Trees (with high flexibility over the data, widely adopted in species distribution models^79^.

#### Model construction and evaluation

To ensure the spatial independence assumption between training and testing dataset, the original dataset was separated into five folds using the BlockCV R package^80^ which is very useful to avoid close points from being in testing and training dataset at once^81^. The block size was set at 250 km after a preliminary analysis showing that it was the minimum distance to assume spatial independence (details are provided in the supplementary material). A total of 5 models for each algorithm were fitted on 4/5 of the data, then evaluated on the remaining 1/5. For each model, True Skill statistic, Area Under the receiver-operator Curve, optimal TSS threshold, Specificity and Sensitivity were calculated as evaluation of model performance^82^.

To evaluate both the models and the stability of the results, a bootstrapped cross-validation was chosen use the advantages of cross-validation as a mean to test model performance on independent data, and bootstrapping which allows to estimate the variation in these performance metrics^83^. fivefold cross-validation was conducted for the two algorithms 30 times with a different random spatial fold assignment. This resulted in 2 × 5 × 30 = 300 models. Each model constructed was fitted with 4/5 of the data and then evaluated with the remaining 1/5. For each model: (1) The AUC, TSS, specificity and sensitivity and optimal TSS threshold are calculated, (2) a current-day and future prediction layer are calculated, from which are derived binary maps (0/1) using the calculated threshold, and (3) variable importance is calculated by random permutation for each predictor value in the prediction dataset, and is the associated loss in AUC in the permuted prediction, finally (4) response curves are calculated for each model.

#### Ensemble modelling

The models in the first quartile of AUC (Hence the 25% poorest models) are discarded to avoid including models fitted on inefficient data to accurately capture habitat suitability. The remaining 75% (225 models) are used in the ensemble predictions. The ensemble habitat suitability map is the mean of all binary predictions (0/1), as the individual model habitat suitability may be biased by algorithm types. The ensemble binary threshold is calculated by evaluating the ensemble prediction on the whole aggregated dataset, deriving an optimal threshold maximizing TSS using the presenceabsence R package^84^.

### Model uncertainty

Uncertainty assessment was estimated using three separate methods: (1) Calibration uncertainty was calculated by dividing the mean output of the ensemble modeling by its standard deviation, resulting in the relative variation in output by grid cell, which allows us to see areas where disagreement between models is higher depending on the spatial folds assignment; (2) Algorithm uncertainty by applying the ensemble modeling methodology separately for retained GAMs and retained RFs, to highlight where algorithms disagree in present and future conditions; and (3) Model-Observation disagreement, by comparing current-day suitability with observed presence areas. The latter is computed by using ordinary Kriging on survey data (Full dataset without pseudo-absences) with a distance buffer of 20 km, allowing us to see where suitable areas overlap or contradict the survey data.

### Climate change projections and fishery impact analysis

Predictive habitat suitability maps were produced using present-day environmental predictors, while future climate change predictions according to the RCP 8.5 2100. Bottom temperature, salinity and current velocity were switched for their predicted values under climate change. Two habitat suitability maps of *I. elongata* were obtained for present and future conditions. Both maps were converted to presence-absence and then combined to identify four areas: (1) Habitat gain; (2) Habitat loss, when the area is suitable today but not in the future; (3) full absence, and (4) *climate refugia* or refugia, when the habitat is suitable at present and in the future.

Successively, the risk of impact from bottom trawling fisheries was estimated based on Fishing hours by km2 of European trawlers for the 2012–2020 period, derived from processed Automatic Identification System (AIS) data by the Global Fishing watch database^73^. The AIS layer was discretized into four risk level based on quartile distribution. “Low” ranging from 0.01 to 1.35 h, to “Medium” between 13.5 and 4.47 h, “High” between 4.47 and 17.58 h, and “Very high” for values higher than 17 h.

This layer was used independently from the model, as it overlapped with present (interpolated), potential (suitable habitat in present conditions) and refugia (predicted persistent future habitat suitability).

### Ethical approval

The authors have received permission to access and use the data presented in this study. All surveys were conducted following relevant national and European regulations, and all protocols were validated by the General Fisheries Commission of the Mediterranean Sea of the Food and Agriculture Organization of the United nations which curates the VME database.

## Results

### Model performance

The mean AUC of the cross-validation was 0.82 + /− 0.06, the TSS of 0.58 + /− 0.9, Sensitivity of 0.92 + /− 0.01, Specificity of 0.71 + /− 0.1. These metrics show a good model fit, with an excellent sensitivity and acceptable specificity i.e. with models who are better at identifying presences than absences.

### Environmental variables contribution and response curves

Our results indicated that bathymetry was the most contributing environmental predictor for *I. elongata* habitat selection, with an optimum around 600–700 m, while low habitat suitability was indicated at shallower depths. The response curves of probability of presence of *I. elongata* indicate a less suitable habitat below the optimum, but RandomForests tends to still consider depth below −1000 m to be suitable, when GAMs do not.

**Figure 3:**
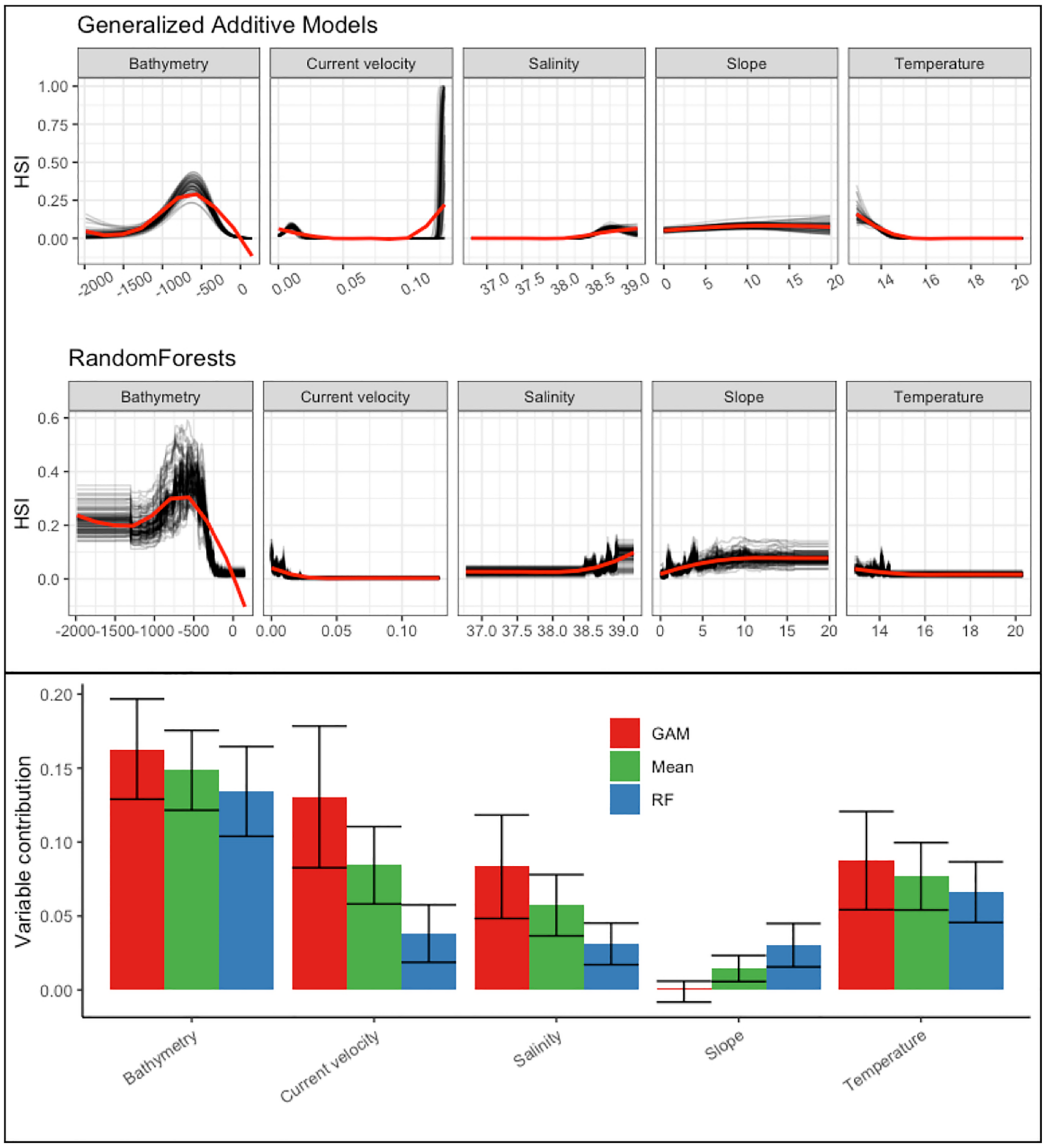
Modelled predictors variable summary Top: Response plots by algorithm, with black lines representing the retained model response and the red line showing a LOESS estimate of the mean response for visualization purpose. Bottom: Mean and standard deviation of predictor contribution to retained models by algorithms. Created using ggplot2 version 3.4.4 https://cran.r-project.org/web/packages/ggplot2/index.html within the R environnement (R Core Team^100^).

Current velocity seems to be a powerful predictor for GAMs, with a shared optimum between both algorithms, around 0.01 m.s^−1^, although some uncertainties remain for extreme values of this environmental factor (> 0.1 m.s^−1^) as shown in Fig. 3. Both GAMs and Random Forest agree on an optimum at 38.8 psu of salinity. In contrast, slope shows a marginal effect on the habitat selection of *I. elongata* in RandomForest and seems almost unrelated to it in GAMs. In general, both models favor slightly medium to high values of slope (> 3°) (Fig. 3). A negative curvilinear relationship is shown for temperature in both models, especially in GAMs where this effect is more marked.

### Isidella elongata habitat suitability maps

**Figure 4:**
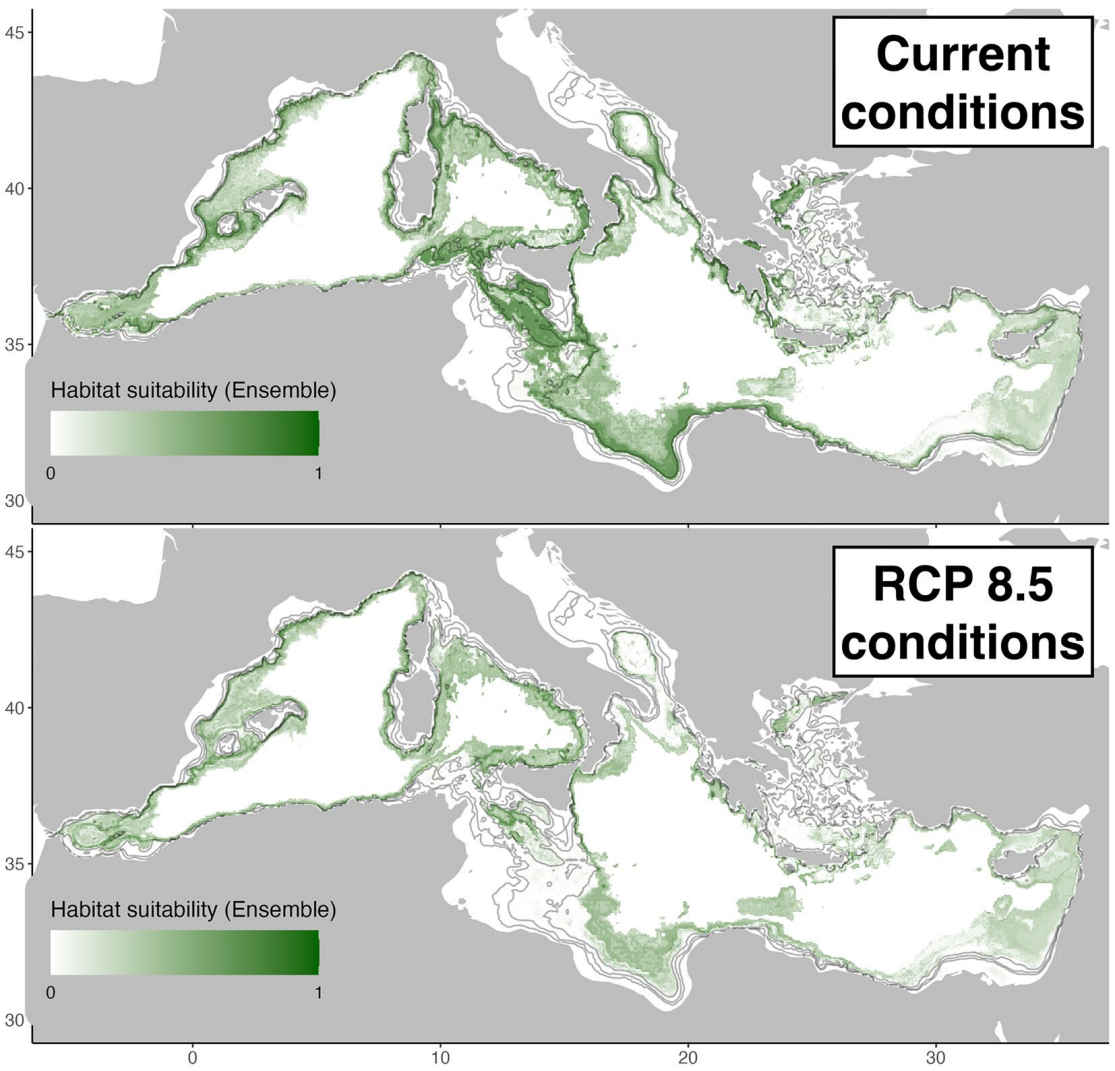
*Isidella elongata* habitat suitability index from the ensemble model at present day (top) and 2100 (bottom) according to the RCP 8.5 IPCC scenario. Created using ggplot2 version 3.4.4 https://cran.r-project.org/web/packages/ggplot2/index.html within the R environnement (R Core Team^100^).

**Figure 5:**
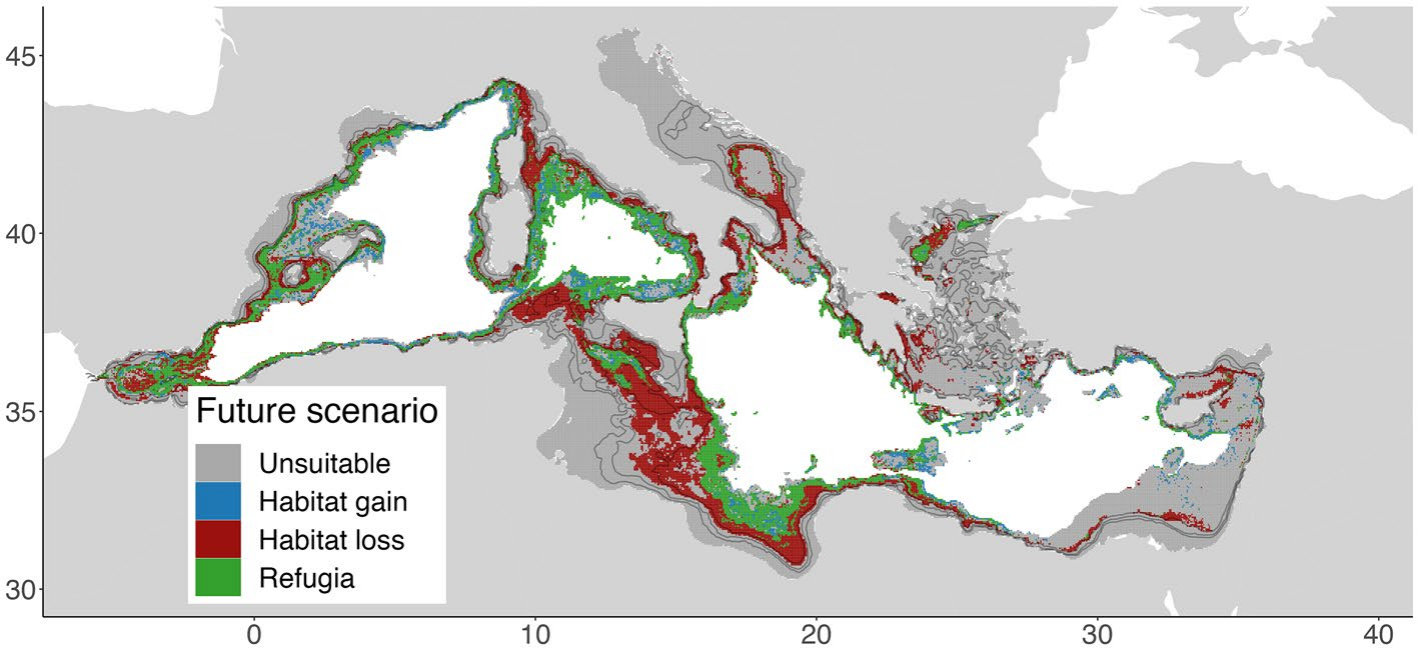
Suitable habitats evolution of *Isidella elongata* in the Mediterranean Sea as predicted by the ensemble model for the expected climate conditions in 2100. Created using ggplot2 version 3.4.4 https://cran.r-project.org/web/packages/ggplot2/index.html within the R environnement (R Core Team^100^).

Under current climate conditions, the habitat suitability of *I.elongata* is widespread in the continental slope of the Mediterranean, with higher probability of presence in the central Mediterranean such as the Strait of Sicily, Tyrrhenian and Ionian seas. The climate projection shows a decrease in habitat suitability more accentuated in the eastern and central Mediterranean, while the habitat suitability more stable in the western basin, the southern Ionian, south Adriatic and Strait of Sicily are noticeably the areas with the largest portions of habitat loss. Conversely, areas of habitat suitability gains are mostly found in deeper waters below 1000 m, in the western and eastern basin, and habitat loss in the shallower part of the initial habitat suitability (Figs. 4 and 5).

### Uncertainties and model error

**Figure 6:**
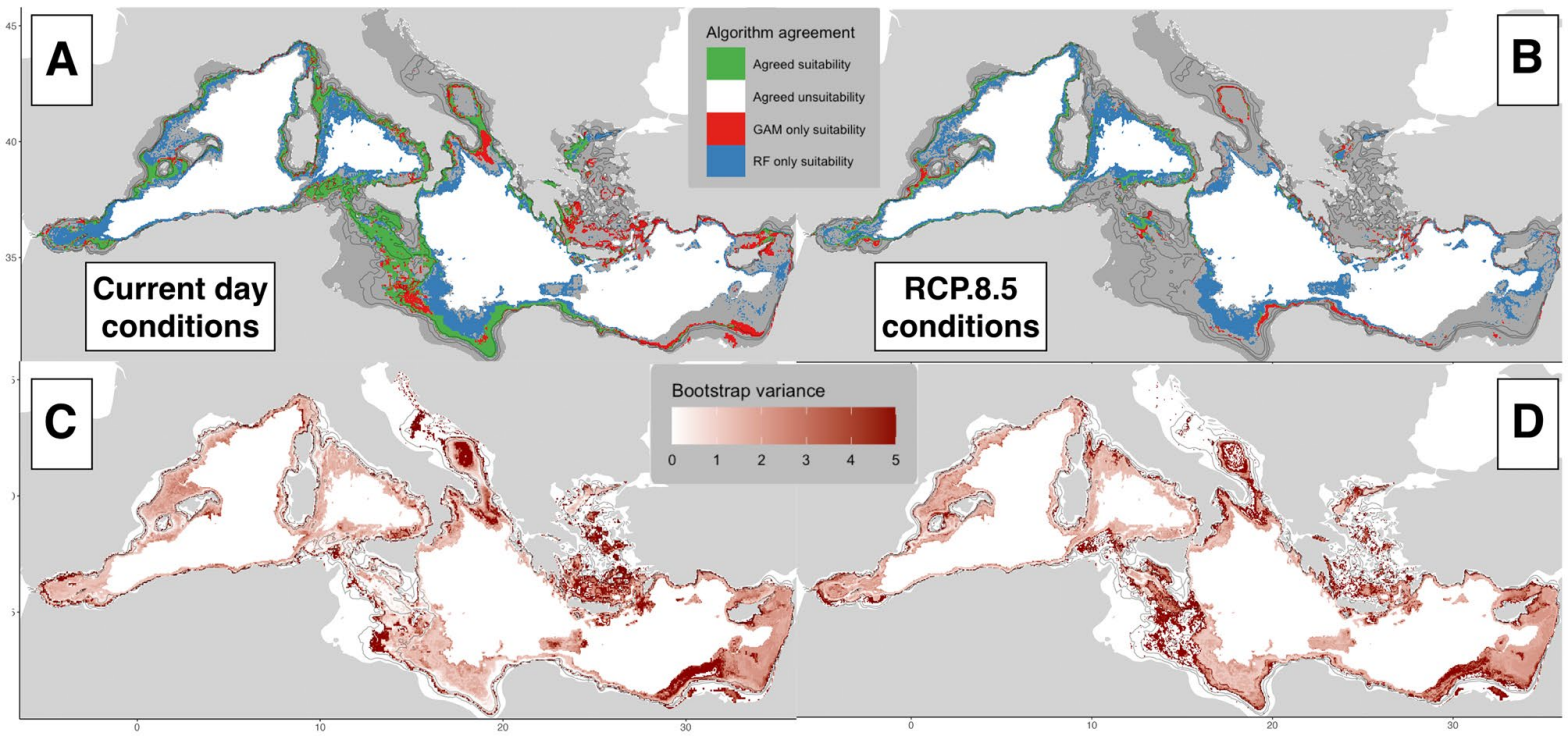
Uncertainty maps of the ensemble model, by algorithm disagreement (**A**, **B**) and calibration uncertainty (**C**, **D**), for current conditions (left column) and RCP 8.5 (right column). Created using ggplot2 version 3.4.4 https://cran.r-project.org/web/packages/ggplot2/index.html within the R environnement (R Core Team^100^).

Calibration variance highlights areas where habitat suitability varies depending on the model construction. Under current environmental conditions (Fig. 6c), this uncertainty is higher in the Aegean Sea, south Levant, southern Strait of Sicily, and mid-southern Adriatic Sea. It is also consistently high below 1000 m depth. Future predictions suggest that it is the most in the Strait of Sicily, Otranto channel in the southern Adriatic and south Levant Sea (Fig. 6d), but remains quite stable relatively in the western basin, while conserving high uncertainty below 800 m.

Both algorithms agree in areas between 200 and 800 m depth range and in western and central Mediterranean (Fig. 6a,b). The disagreement between both models is stronger in deeper water, with RandomForests considering areas deeper than 1000 m still adequately suitable for *I. elongata* habitat selection while GAMs classify them as unsuitable habitat. In shallower waters however, both algorithms show a good agreement in the future probability of presence suggesting that the habitat will not be suitable above 600 m depth. The difference between future projections in various areas regards the eastern basin, with GAM favoring the habitat suitability of the southern Aegean, while RandomForest in the Levant Sea.

**Figure 7:**
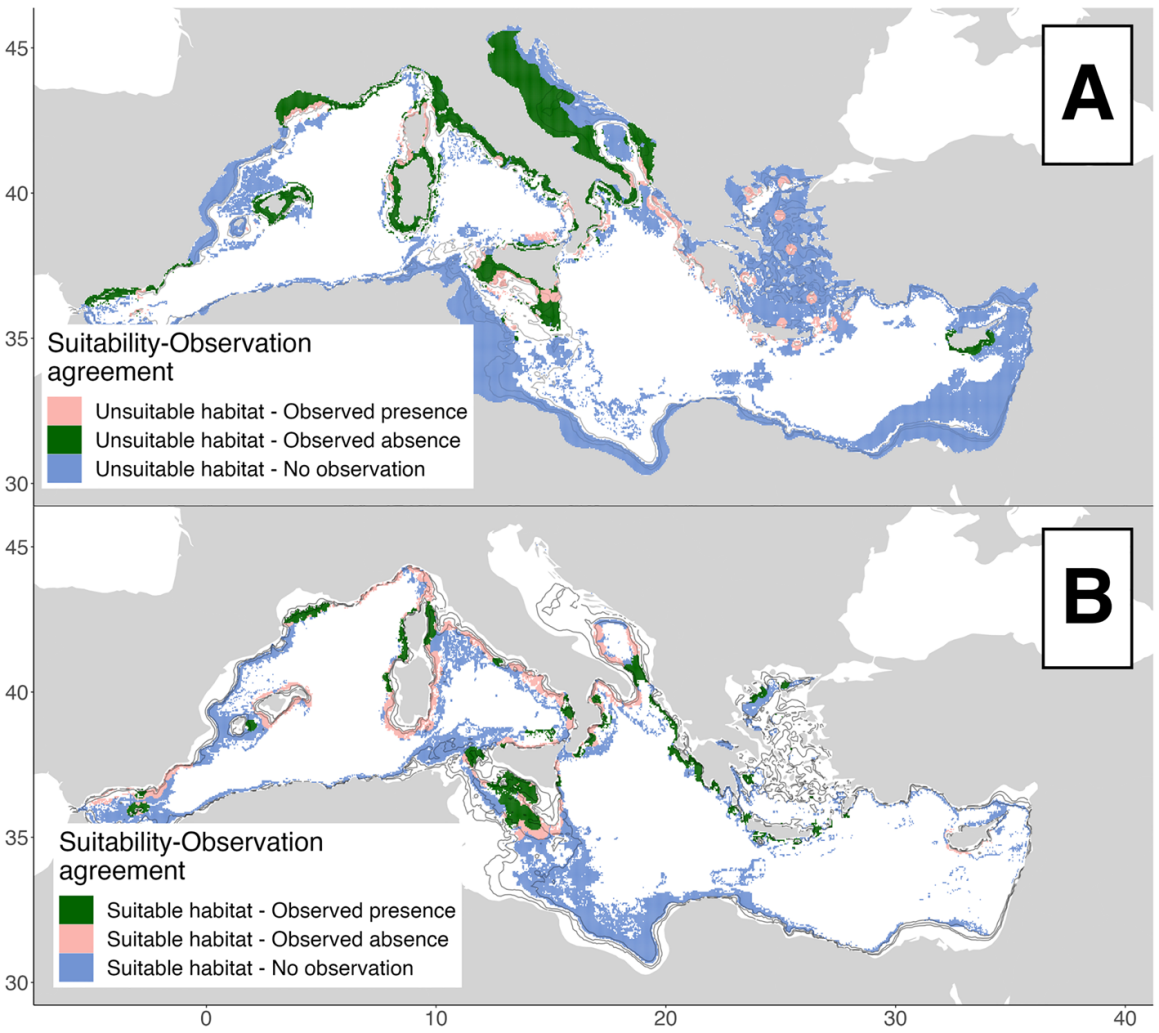
Comparation between modelled suitability and interpolated occurrence, for (**A**) suitable habitats and (**B**) unsuitable habitats. Green represents agreement, red disagreement and blue surveyed areas. Created using ggplot2 version 3.4.4  https://cran.r-project.org/web/packages/ggplot2/index.html within the R environnement (R Core Team^100^).

Figure 7 represents the agreement between suitable habitat and observed distribution of the species. Our results suggest a good match in the Strait of Sicily, North-east Ionian, Gulf of Lion, south Tyrrhenian Sea and Corsica. Differently a mismatch is found in the Tyrrhenian, Ligurian sea, Sardinia Sea and in the Aegean Sea (Fig. 7a). Regarding the unsuitable habitat prediction, our models output shows an agreement in most of the surveyed areas for absences, while mismatch is mostly bordering observed presences areas (Fig. 7b).

### Fishing risk analysis

**Figure 8:**
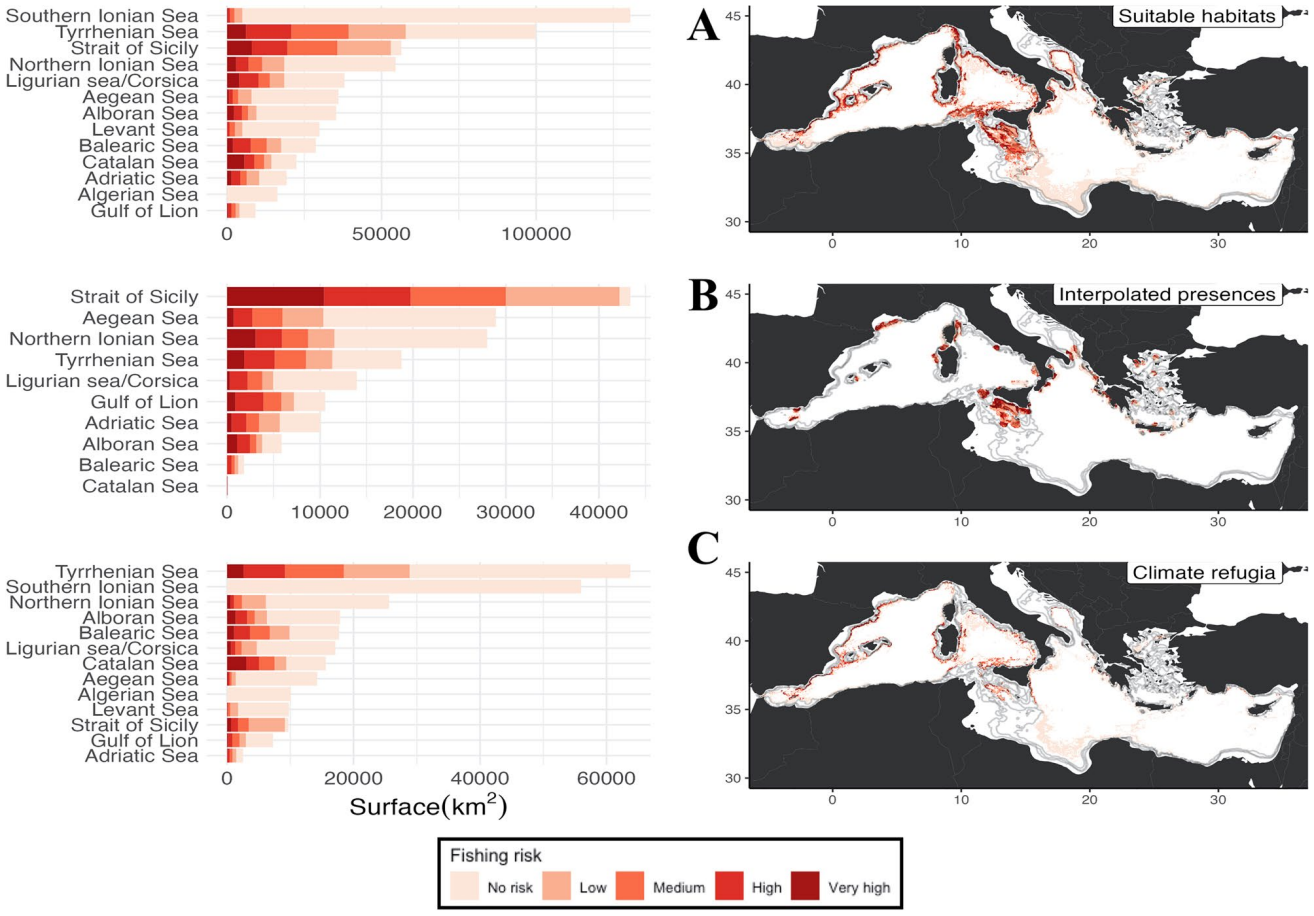
Overlap of fishery impact analysis on observed presence (**A**), predicted habitat suitability (**B**) and predicted climate refugia at 2100 (**C**), with (left) barplots of total surface by risk level in regional seas. Created using ggplot2 version 3.4.4 https://cran.r-project.org/web/packages/ggplot2/index.html within the R environnement (R Core Team^100^).

Fishing risk analysis shown In Fig. 8 highlights different trends when considering areas of suitable habitats (Fig. 8a), interpolated presences (Fig. 8b), and *climate refugia* (Fig. 8c). In the western basin, trawling grounds overlap with suitable areas, c*limate refugia* and observed *Isidella elongata* grounds, mostly in the Gulf of Lion, Balearic Islands and Catalan Sea. In the central Mediterranean, the Tyrrhenian, Sardinia Corsica, and Strait of Sicily count as the main surfaces, with a stress on the latter being the first areas in terms of overlap with observed presence of overlap. In the eastern Mediterranean, the North-East Ionian, and Aegean suitable habitats and observed presences also overlap with trawler activity.

## Discussion

This study enhances our understanding of the habitat requirements and future climate change and fisheries impacts on the critically endangered deep-sea coral *Isidella elongata*. Such results may be relevant for the conservation of VMEs associated with this habitat-forming species under the protocols for the protection of VMEs in application of the GFCM/43/2019/6 resolution^22^, as well as prove useful for the Mediterranean countries willing to implement measures for threatened deep-sea corals following the Barcelona Convention^23^.

### Environmental variables and predictive distribution map

Our results suggest that *I. elongata* suitable habitats are located on the continental slope, in a depth range below 200 m, with an optimum of 700 m at Mediterranean scale. Several regional studies have described the preference of this species for certain depths that vary with the specific characteristics of each area. In the central and western Mediterranean Sea, *I. elongata* has been observed at depths ranging from 232 to 1308 m^5,9,10,26,38^ 350–670 m in the Gulf of Lion^40^ but also at 1730 m depth (Fabri et al. unpublished data) or between 200 and 800 m depth in the Strait of Sicily^7^ In the Aegean Sea this species can be found at depths between 126 and 1125 m^﻿8^ and also occasionally in shallower waters. Even though some findings might be influenced by the data availability, and the precise suitability below 1000 m meters is unclear, the overall depth range where *I. elongata* is likely to occur is wide and in some areas overlaps with areas of intense bottom fishing activity.

Sea bottom temperature and salinity also appear to play a significant role in the spatial distribution of *I. elongata*, with an optimal temperature below 13.75 °C and salinity around 38.7 PSU. Except for the Gulf of Lion and the Otranto Channel where the seasonal thermocline may reach the upper boundary of I. elongata distribution range during winter convection, the rest of its distribution is located below the thermocline where temperature is stable^85^ It is important to state that lower salinity is likely not a limiting factor as is for the species since the species may be found sporadically in the Atlantic.

Although this species is generally found on low-slope substrata^15,86^ which are areas of sediment accumulation typical of flatter areas, our results suggest that a positive relationship occurs between the probability of presence of *I. elongata* and moderate value of slope in the Mediterranean Sea. This type of habitat preference has been also suggested in the Strait of Sicily where *I. elongata* did not seem particularly associated with low slope steepness, probably because steeper areas are shelters from bottom-trawling^7^ The relatively low power of slope in our models may be explained by the missed finer-scale complexity of topography and oceanographic processes (open slopes versus canyons) making it difficult to draw a basin-wide trend on this parameter^87^. Furthermore, Cartes et al. (2013)^26^ stated that in the western basin, high near-bottom zooplankton biomass reached a peak between 1000 and 1300 m, where temperatures are low and can be associated with large populations of *I. elongata*. Our models suggest a habitat preference for low to moderate current velocity regime, although it is unclear if this is linked to food availability or substrate preference. Overall, a combination of temperature, salinity, and current preferences of this species may represent an optimum for food availability, although more knowledge is required to precisely describe it.

The modeled habitat suitability confirms that continental margins are the most suitable habitats for *I. elongata*. This niche corresponds to shelf breaks where trawling is difficult due to the complexity of the seafloor, with microhabitat topography features offering protection for this species from fisheries impacts. Bo et al. (2015)^88^ described a pristine *I. elongata* colony at 210 m depth on the south-west coast of Sardinia in an area not impacted by fisheries because of the presence of rocky substrate. Furthermore, it is well known that *I. elongata* was present at shallower depths in the past, but the increasing depths where bottom trawling occurs has durably changed this paradigm. González-Irusta et al. (2022)^89^ has shown how fisheries may have induced loss of habitat of this species, by comparing the realized niche of the species with the estimation of its past distribution before trawling.

Our results show that the ensemble habitat suitability model performs well for the central Mediterranean where there is a good agreement with this species' observed records and habitat suitability. Areas of high suitability were predicted in the northern Ionian Sea and southern Adriatic (Otranto channel), in the Strait of Sicily, on the continental margins of eastern Corsica and Sardinia islands, in the eastern Tyrrhenian Sea. These results are coherent with several case studies in literature^6–8,10,10,15,90^. However, generally (i.e., Adriatic Sea, Tyrhennian, Ligurian), the predicted suitable habitat areas were larger than the observed presence areas. In the western basin, suitability is ubiquitous in the suitable depth range and encompasses most of the recorded presence areas (Gulf of Lion, Balearic Islands and Alboran Sea). Local suitable areas have been found in the Gulf of Lion, with a pattern of presence in canyons with no trawling scars^86^ or in the Balearic Islands, where healthy colonies are present in areas where trawling is prohibited due to the presence of underwater cables^27^. This area is known as an important area for *I. elongata* historically^91,92^. Finally, the results are more uncertain in the southern Mediterranean and eastern basin, which is probably linked to lack of sampling effort, which is a recurring problem in these areas.

### Climate predictions and identification of refugia

Our results under the climate change RCP 8.5 scenario showed high habitat loss in the central Mediterranean, the expansion of suitable areas for *I. elongata* is much lower than the predicted habitat loss. About 60% the currently suitable areas for *I. elongata* are no longer forecasted in 2100, most of which are in the central basin. This change seems to be mainly driven by the sea bottom temperature anomaly predicted for 2100 (Fig. S6 Supplementary material) that suggests a rise of 1–2 °C across all Mediterranean Sea continental margins. Still, the western basin being relatively colder than the eastern one, future temperatures are forecasted to reach the current temperatures of the eastern basin and suitable thermic areas (below 14° C optimum for *I. elongata*) are found to have generally shifted westward. In contrast, bottom salinity and current velocity anomalies are less marked at the depth range where *I. elongata* is found, making their contribution less important. This effect of temperature could be related to the fact that *I. elongata* lives in deep-water ecosystems which are quite stable over time (i.e. low variability in water properties) compared to shallower ecosystems and that climate change is expected to affect the fitness of this species in response to a higher temperature^50,93^.

Across the bathymetric gradient, the loss of suitability is the strongest above 600 m, while most high confidence refugia are within the 600-1000 m range. The deeper suitable areas are associated with lower sampling effort, and high model uncertainty, preventing us from making inferences on these depths, although is it likely that local conditions may allow *I. elongata* to strive in some deep areas, like seen in the Aegean sea and gulf of Lion as (Fabri et al. Unpublished data)^8^.

Effects of climate change follows a longitudinal gradient with warming more intense in the eastern and central basin, resulting in habitat loss more marked in these areas in the 600–1000 m range. Although the uncertainty on the forecasted climate refugia may be high, their distribution still reflects areas of oceanographic stability that are important to acknowledge for long-term conservation purposes. The general trend of habitat suitability evolution indicates a shift of the suitable areas for *I. elongata* towards deeper waters. Despite that, not much is known about the environmental conditions associated with these areas.

### Fishery impact analysis and conservation implications

Trawling activity has been more intensive and persistent during the last century in the western Mediterranean where *I. elongata* colonies are scattered over small, localized areas. This reflects that its distribution may not only be limited by oceanographic conditions, but also by an underlying disparity in historical fishing activity. This hypothesis is quite difficult to verify as it would require spatially precise quantification of trawling pressure on the seabed, based on reliable vessel tracking, for example, the Vessel Monitoring System (VMS) used in EU countries, and gear dimension information. However, this kind of data collection program only started at the beginning of this century, while deep bottom fisheries started being economically viable in the second half of the twentieth century^94^. Moreover, VMS data are often difficult to access due to data privacy limitations^74^. González-Irusta et al. (2022)^11^ showed using SDMs, the habitat reduction of *I. elongata* grounds imputable to trawling intensity and supporting the hypothesis of a dramatic effect of half a decade of trawling^95^. The current distribution of the bamboo coral reflects the overlap between its overall habitat suitability and trawling footprints. However, it is important to note that trawling pressure has shown important changes during the last years, with a significant decrease in the western Mediterranean, more than 25% between 2015 and 2020, and that could reach up to 40% by 2025, following the multiannual plan for the fisheries exploiting demersal stocks in the western Mediterranean sea. This reduction of the current and future trawling effort and potential changes in the distribution of their effort has not been considered in this work and thus their role in the potential recovery of the species is still unknown.

The results of the fishery impact analysis compare the overlap between fisheries and observed presences, predicted presences, and climate refugia respectively. This allowed the identification of areas in need of urgent protection and appropriate fishery management plans, mainly the Strait of Sicily, the South Tyrrhenian Sea, the North-east Ionian, and the Gulf of Lion hosting large documented coral gardens.

In this regard two conservation strategies may be considered: the first could focus on the conservation of *climate refugia* most threatened by trawling efforts, where trawling may be banned to allow the recovery of *I. elongata* colonies such as the continental shelves of the Strait of Sicily, the eastern Ionian Sea, Otranto channel and Tyrrhenian. The second strategy would aim to the protection of less-fished climate refugia which still host healthy colonies and are the most likely to survive the next century. In this case, the western Mediterranean Sea could be a potential area where to implement such protection. However, it is also worth stressing that if some *I. elongata* facies have been not impacted by fisheries because the current technological limitations of trawling, and it is not certain that this protection will persist as trawling gear technology advances and currently exploited areas become less profitable, inducing the development of novel fishing strategies toward new fishing grounds.

### Study limitations and caveats

Despite this work aiming to predict the habitat suitability of *I. elongata* at Mediterranean scale we acknowledge the fact that this task is hindered by some limitations. This deep-sea octocoral species has been severely impacted over the last decades by bottom trawling, therefore the spatial distribution that we are observing is limited by both the environmental conditions and fishing impact, thus current *I.elongata* absences areas may be linked to complete removal by trawling, influencing the response of the models on all variables^89,96^. This is indicated by the strong overlap between trawlers activity and suitable habitat as shown in the fishing risk analysis. Our results suggest that the retained parameters relations and predictive power shows an adequate explanatory power of the theoretical niche of the species in the Mediterranean Sea. Still it is possible that the mis-match between habitat suitability and actual distribution in some areas might be due to a wide spectrum of non-environmental variables such as biotic interactions, larval dispersal and other environmental parameters not considered missed in this study.

Another important factor that could not be included in the model construction was calcite saturation, which is a key parameter in biocalcification and that is expected to evolve with climate change, affecting the fitness of the species. However, we were not able to find a reliable environmental layer adequate to the scale of the study, which provided good predictive power along with an ecologically sound response. It is worth noting that the current Mediterranean is generally supersaturated in calcite^97^, therefore it is possible that climate change will have a more limited impact on this factor comparing to other oceans and other environmental parameters such as temperature. This is also a factor to keep monitoring, since de-saturation increases with depth, it acts in the opposite direction of temperature, which is decreasing with depth, possibly inducing a shrinking of the suitable condition windows for these two key parameters.

As for the AIS data (used as trawler activity proxy) this was the only publicly available data at Mediterranean scale, and it is mandatory only for European vessels above of a Length overall above 15 m. However, it calculates fishing time from speed profiles calculation which may result in mis-identification and satellite coverage, as well as being highly underestimated in the southern Mediterranean where its use is not compulsory. However this is not considered a problem in this study as *I. elongata* is most at risk from deep water trawlers, where the mean fleet size is above 20 m in the Mediterranean Sea^98^.

## Conclusions

This study has important implications for the conservation of soft- and compact-mud VMEs in the Mediterranean Sea as it represents the first Mediterranean-wide attempt to identify such key areas on a large scale. Probable habitats of *Isidella elongata* were identified and mapped, but this suitable habitat distribution may change in some areas, mainly in the central Mediterranean Sea, with deep water warming and foreseen changes in water-current regimes. These environmental changes will affect benthic fauna feeding, dispersion, and calcification capabilities, although the processes of these changes remain challenging to model explicitly. Bottom trawling is directly impacting *I. elongata* fields and reducing this pressure, which is already in place in many Mediterranean parts with the application of management plans, can help this ecosystem to recover. Potential management plans could consider a trawling ban at 800 m depth similarly to the Northeast Atlantic (it has entered in force in 2016 as well as a ban on all bottom gears on VMEs below 400 m in 2022). Although the impacts of climate change seem much harder to mitigate, the prevention of fisheries impacts on *I. elongata* fields, where models have foreseen *climate refugia* in 2100, could favor these VMEs resilience in the future. Conservation measures must be discussed for each area where *I. elongata* is known to occur and expected to remain in 2100, as this species provides ecological benefits to the deep-sea trophic network and indicates the good environmental status of deep-sea ecosystems. The upcoming research effort in the long-term should be directed to integrate and summarize multiple threats^61,99^ and their effect on multi-VMEs indicator species to provide a comprehensive snapshot of the status of marine ecosystems to policymakers and push for truly efficient regional management measures linked to current and future fishing management measures to conserve these species and their associated habitats.

### Supplementary Information

Supplementary Information.

## Data Availability

The occurrence dataset was constructed partially from the GFCM VME complete database, for which the access is partially restricted, thus the authors cannot share as it is. The MEDITS data is available at the discretion of each area’s MEDITS coordinator. The ROV surveys come from the GFCM VME database and thus may or may not be open access. We kindly invite any interested parties to contact the corresponding author with their areas/survey of interest, so that they can be directed to the appropriate data curators, to which they will need to formally request the data. The output layers are available upon reasonable request to the corresponding author.
