# Supplementary material for: Mapping the habitat refugia of Isidella elongata under climate change and trawling impacts to preserve Vulnerable Marine Ecosystems in the Mediterranean
Source: Sci Rep. 2024 Mar 15;14:6246. doi: 10.1038/s41598-024-56338-1 (PMC10940633; doi:10.1038/s41598-024-56338-1)
Supplement: Supplementary file 1 — Supplementary Information. [file 41598_2024_56338_MOESM1_ESM.docx]

**Supplementary Material of the article: Mapping the habitat refugia of *Isidella elongata* under climate change and trawling impacts to preserve Vulnerable Marine Ecosystems in the Mediterranean**

**Occurrence dataset**

- Data sources
- Temporal distribution
- Depth distribution

**Environmental Predictors**

- List of predictors considered in the study
- Correlation analysis
- Observed *Isidella elongata* records and statistical metrics for retained predictors
- Maps of present and future environmental conditions

**Data preparation**

- Pseudo absence implementation
- Spatial block size determination

**Model performance**

- Evaluation metrics values for the constructed models

Table S1: Data sources and time frame of the datasets used for this study.

| Survey name | Type of survey | Data sources | Time frame | Number of data points |
| --- | --- | --- | --- | --- |
| MEDITS | Bottom trawling survey | Ministry of Agriculture, Rural Development and Environment, Cyprus | 2004-2020 | 4940 |
|  |  | Centro Interuniversitario di Biologia Marina ed Ecologia Applicata University of Bologna, Italy |  |  |
|  |  | University of Bari, Italy |  |  |
|  |  | COISPA Tecnologia & Ricerca, Italy |  |  |
|  |  | University of Cagliari, Italy |  |  |
|  |  | Hellenic Centre for Marine Research, Greece |  |  |
|  |  | Instituto Español de Oceanografia (IEO-CSIC), Spain |  |  |
|  |  | Institute for Biological Resources and Marine Biotechnologies, CNR IRBIM, Italy |  |  |
|  |  | Ministry for agriculture of Malta  Department of Fisheries and Aquaculture,  Fishery research unit |  |  |
|  |  | MARBEC, Ifremer, France (Jadaud & Certain 1994) |  |  |
| NOURMED |  | Ifremer, France (Vaz, 2018a) | 2018 | 102 |
| SECO DE LOS OLIVOS/BALEARES | ROV surveys | Aguilar R, GFCM Database on VME (Unpublished) | 2010 | 43 |
| CORSEACAN |  | France (Fourt & Goujard, unpublished) GIS posidonie | 2010 | 89 |
| MEDSEACAN |  | France (Fabri et al. 2014, 2020) | 1995-2010 | 112 |
| AEOLIAN |  | Aguilar R, GFCM Database on VME (Unpublished) | 2018 | 178 |
| LIFE BAHAR MALTA |  | LIFE BaHAR for N2K | 2015-2016 | 276 |
| RESHIO | Fisheries dependent and independent surveys | Gerovasileiou V, Smith C, Mytilineou C, HCMR-Hellenic Centre for Marine Research (unpublished) | 2000-2001 | 30 |
| INTERREG |  | Gerovasileiou V, Smith C, Mytilineou C, HCMR-Hellenic Centre for Marine Research (unpublished) | 1999-2000 | 16 |
| CORALFISH | Longline survey | Gerovasilieiou V, Chimienti G , HCMR-Hellenic Centre for Marine Research (unpublished) | 2010 | 12 |
| Gerovasileiou et al 2019 | Diverse | Gerovasileiou et al. 2019 | 1891-2018 | 20 |


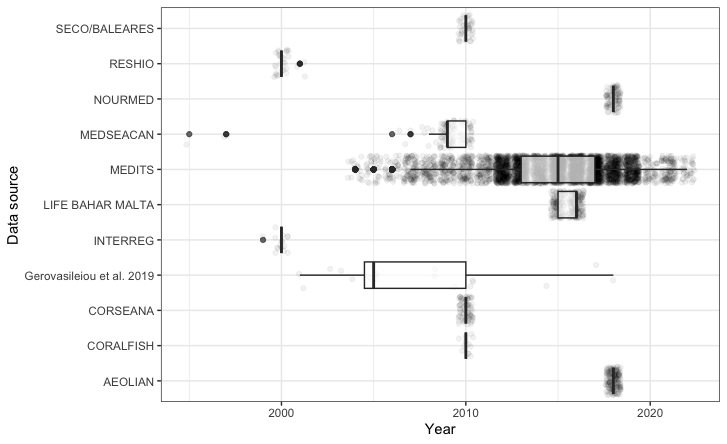


Figure S1: Temporal distribution of points relative year of sampling for each dataset used for model construction by data source (excluding 1 record dated at 1891 from Gerovasilieou et al., 2019 for plotting purposes). Made using ggplot2 v3.4.4 https://cran.r-project.org/web/packages/ggplot2/index.html within the R environnement (R Core Team, 2023).


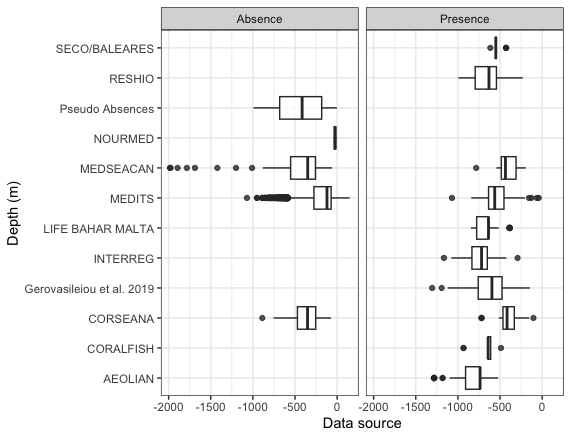


Figure S2: Depth values relative to data points (Presence/Absence) used for modeling construction (extracted from Emodnet Bathymetry) by data source. Made using ggplot2 v3.4.4 https://cran.r-project.org/web/packages/ggplot2/index.html within the R environnement (R Core Team, 2023).

**Environmental predictors details**

Table S2: Environmental and fishery predictors considered in this study.

| Variable | Source | Included in final model | Native resolution | Justification |
| --- | --- | --- | --- | --- |
| Bathymetry | Emodnet (EMODnet Bathymetry Consortium, 2020) | Yes | 0.002 decimal degrees - 100m | Ecological relevance, reliability, predictive power |
| Slope | Emodnet derived | Yes | 0.002 decimal degrees - 100m | Ecological relevance, reliability, predictive power |
| Temperature | Bio-Oracle(Assis et al., 2018; Tyberghein et al., 2012) | Yes | 0.083 decimal degrees - 8 km | Ecological relevance, reliability, predictive power, availability of climate projection |
| Salinity | Bio-Oracle(Assis et al., 2018; Tyberghein et al., 2012) | Yes | 0.083 decimal degrees - 8 km | Ecological relevance, reliability, predictive power, availability of climate projection |
| Current velocity | Bio-Oracle(Assis et al., 2018; Tyberghein et al., 2012) | Yes | 0.083 decimal degrees - 8 km | Ecological relevance, reliability, predictive power, availability of climate projection |
| Calcite saturation rate | (Morato et al., 2020) | No | 3km | Low predictive power, low confidence in the reliability in the Mediterranean sea, eventual limiting factor in the Mediterranean sea,  No coverage in Aegean sea |
| Dissolved oxygen | Bio-Oracle(Assis et al., 2018; Tyberghein et al., 2012) | No | 0.083 decimal degrees - 8 km | High correlation with depth, low confidence on its role as limiting factor for the habitat selection of *Isidella elongata* in Mediterranean waters |
| Rugosity | Emodnet derived | No | 0.002 decimal degrees - 100m | Very highly correlated with slope |
| Primary production | Bio-Oracle(Assis et al., 2018; Tyberghein et al., 2012) | No | 0.083 decimal degrees - 8 km | Correlation with depth |
| Substrate type | Emodnet(Vasquez et al., 2021) | No | NA (Polygon data) | Low confidence in the precision of the layers at the survey depth |
| Fishing effort | Global fishing watch v4 (Kroodsma et al., 2018) | No (but used in post-hoc Fishing risk analysis) | 1km | Lack of coverage for non-EU vessels, low predictive power and dubious response in the models |
| Aspect (East West and North south) | Derived from Emodnet | No | 0.002 decimal degrees - 100m | Low predictive power, low relevance at the scale of the study |

Figure S3: Correlation Matrix of retained predictors. Spearman correlation coefficient of retained covariates, calculated on full study area layers raster layers (Left) and extracted data points values (Right)
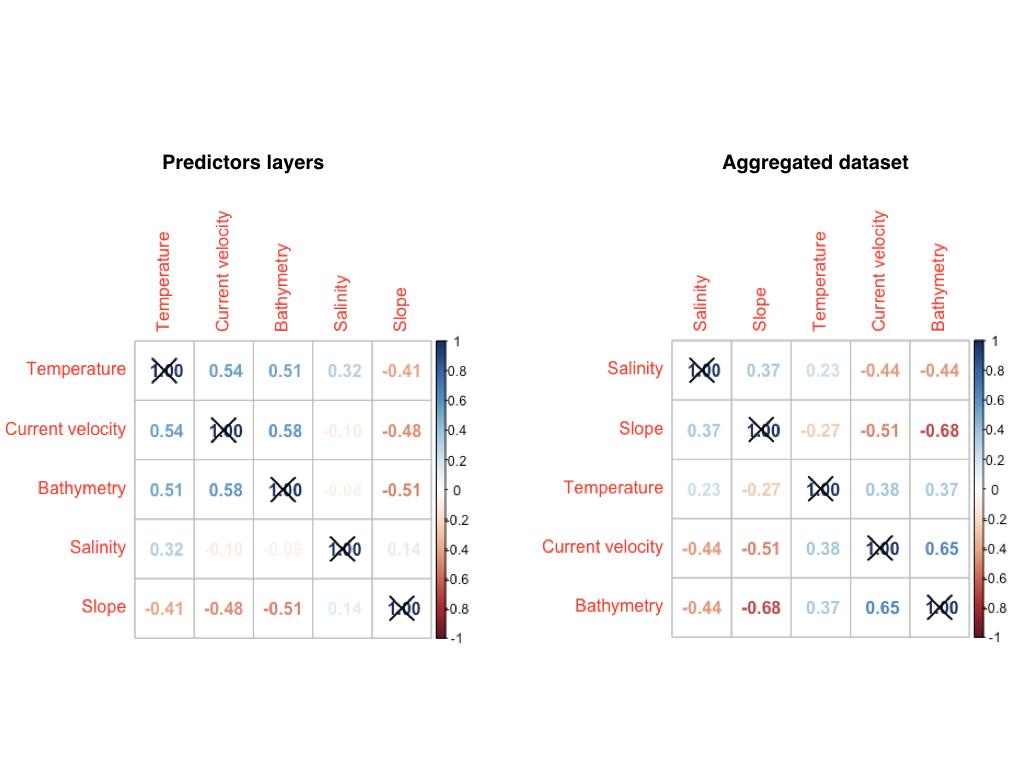


Table S3: Observed *Isidella elongata* records and statistical metrics for retained predictors

| Variable | Salinity (psu) | Slope (°) | Temperature (°C) | Current velocity (m.s-1) | Bathymetry (Meters) |
| --- | --- | --- | --- | --- | --- |
| Mean | 38.67 | 4.92 | 13.70 | 0.0046 | -647 |
| Standard deviation | 0.1 | 3.03 | 0.2 | 0.0024 | 184 |
| 1st quartile | 38.62 | 2.84 | 13.59 | 0.0039 | -736 |
| Median | 38.71 | 4.68 | 13.77 | 0.0042 | -636 |
| 3rd quartile | 38.74 | 5.77 | 13.87 | 0.0056 | -550 |
| Minimum | 38.43 | 0.25 | 13.04 | 0.0002 | -40 |
| Maximum | 38.93 | 15.9 | 14.40 | 0.0200 | -1306 |


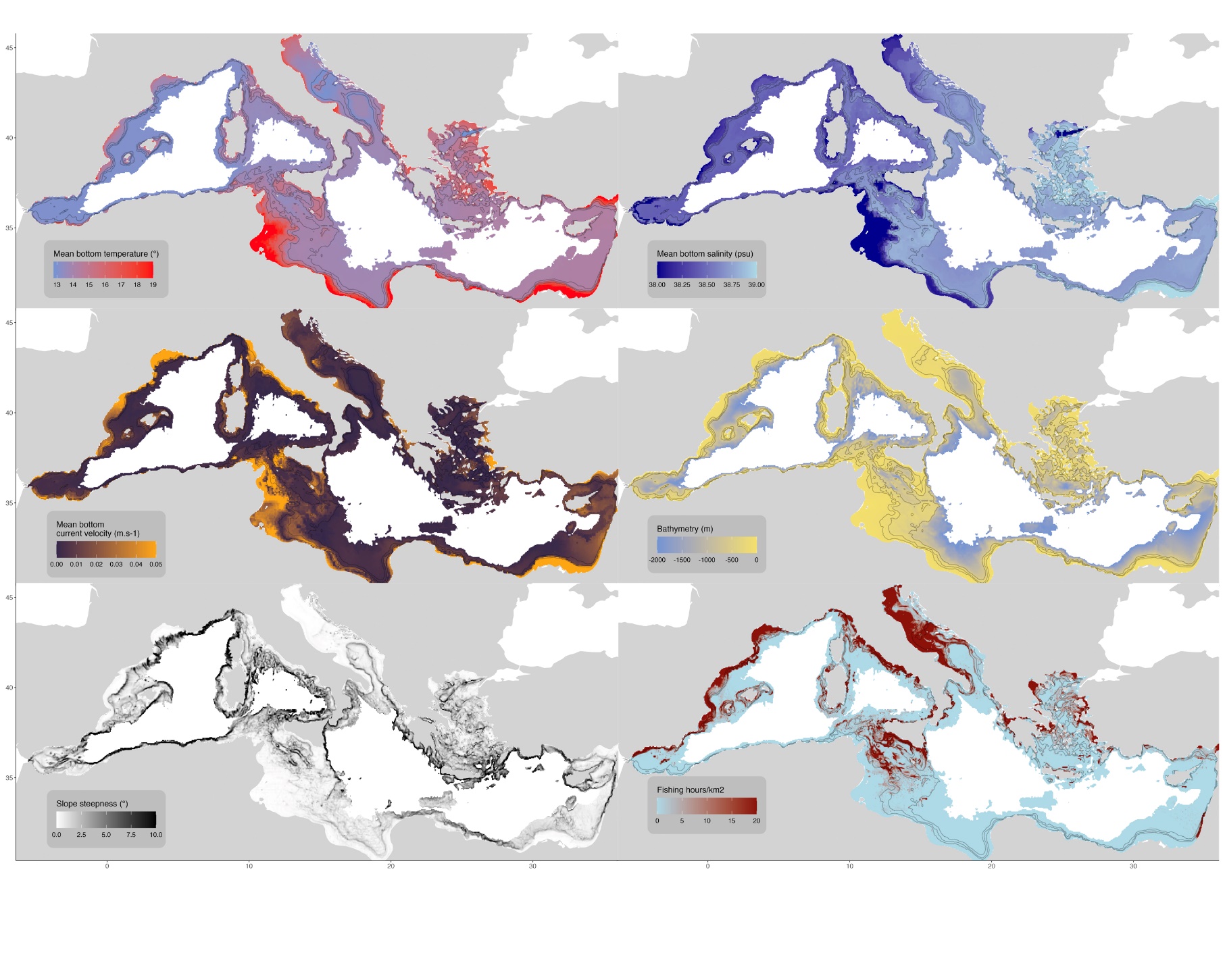
Figure S4: Maps of the environmental parameters and Fishing effort used in the study. Made using ggplot2 v3.4.4 https://cran.r-project.org/web/packages/ggplot2/index.html within the R environnement (R Core Team, 2023).


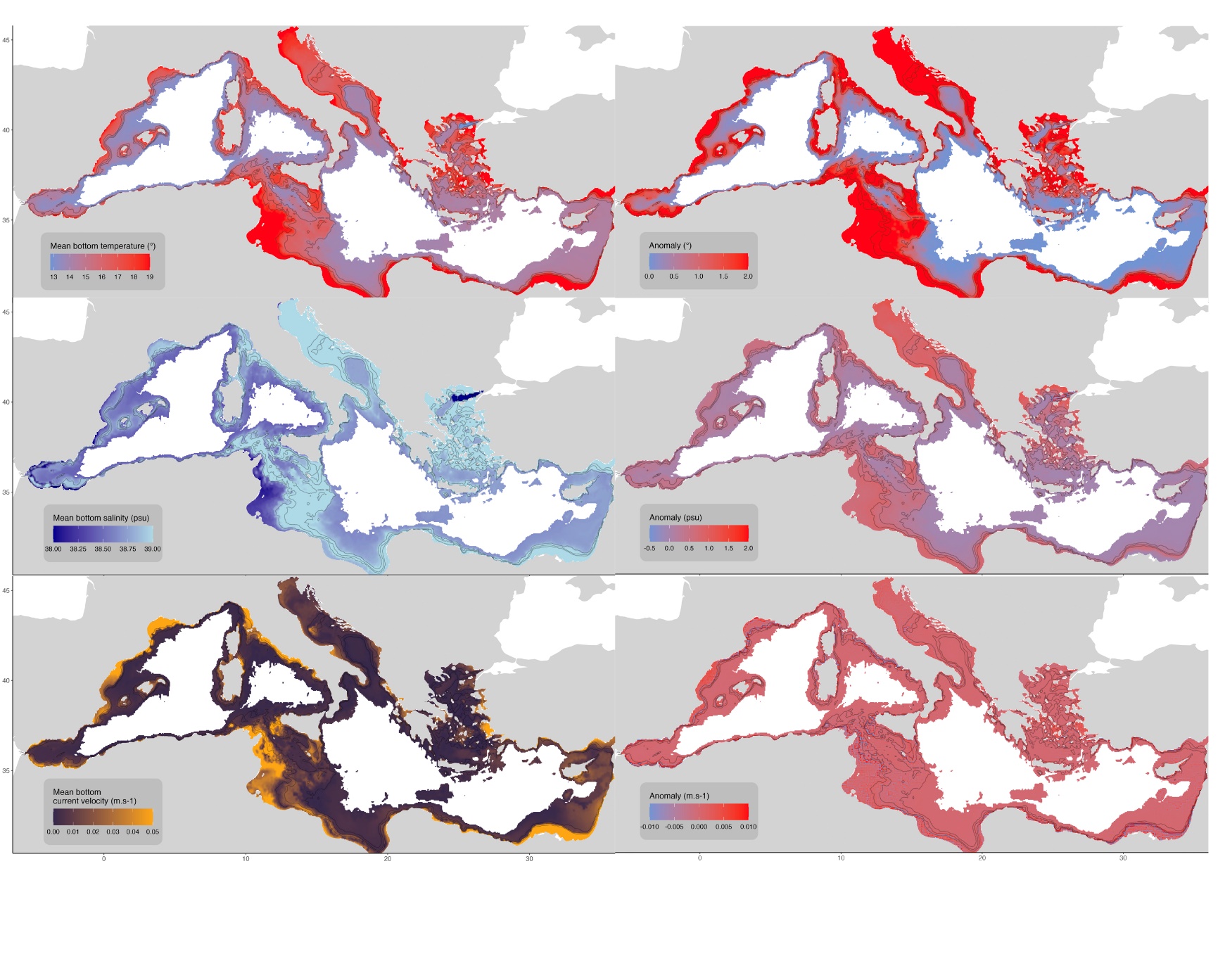
Figure S5: Maps of (Left row) RCP 8.5 climate projection for the 2091-2100 period of the three dynamic environmental predictors used in the climate scenario analysis (temperature, salinity and current velocity), and (Right column) projected Anomaly with respect to the reference period 2000-2014 for these three predictors.

Made using ggplot2 v3.4.4 https://cran.r-project.org/web/packages/ggplot2/index.html within the R environnement (R Core Team, 2023).

**Pseudo absences implementation**

Some areas in the dataset contained a lot of survey presences but no survey absences, due to sampling design and incompleteness of the original data, thus unrelated to the species affinity for those areas. To mitigate this bias, Pseudo absences were implemented in these areas. The pseudo absences were implemented with the purpose to mimic the prevalence (survey presences/absences ratio) in the rest of the dataset.

The whole dataset was classified by depth strata following Medits depths strata (with an aggregation of Strata A and B)

*Strata A : 0-200m,*

*Strata B : 201-500m*

*Strata C = 500-1000m.*

For each of these strata, a mean theoretical prevalence was calculated for all surveys with survey presences and absences. No pseudo absence were implemented below -1000m, due to lack of sampling in these deep areas to make a meaningful relationship.

The calculated theoretical prevalence were:

*All datasets : 0.07*

*Strata A : 0.004*

*Strata B : 0.11*

*Strata C : 0.26*

Two areas were considered: (1) The Aeolian islands in the southern Tyrrhenian sea, and the (2) The north-east Ionian sea and Aegean sea.

**Aeolian islands**

For these small areas, 178 individual presences were observed, but very close to each other, such as when aggregated by 0.041 decimal degree grid cells, we remain with 10 different points. Since the area is small and the pseudo absences is done with 1 points per cell, we were not able to implement the recommended number of 10*(1/0.7) = 143, and were limited to adding 83 points before running out of eligible cells. Nevertheless, this was considered satisfactory to mitigate the sampling bias of this small part of the dataset.

**Northern Ionian sea - Aegean sea**

This area contained a high number of survey presence which could not be dismissed, but to leave as it would bias the model to the conditions of these areas. Pseudo absences were implemented following the procedure, with a number of pseudo absences implemented by strata with the following the formula.

*No of pseudo absences of the strata: No of survey presence * 1/Theorical prevalence of strata*

Strata A :

Observed presences : 3

Number of presences absences : 3*1/0.004 = 611

Strata B

observed presences : 31 :

Number of presences absences : 31*1/0.11 = 282

Strata C

observed presences : 71 :

Number of presences absences : 71*1/0.11 = 276

Resulting in a addition of 1169 pseudo absences in the area.

The pseudo absences and survey presence in these areas are plotted in Figure S6


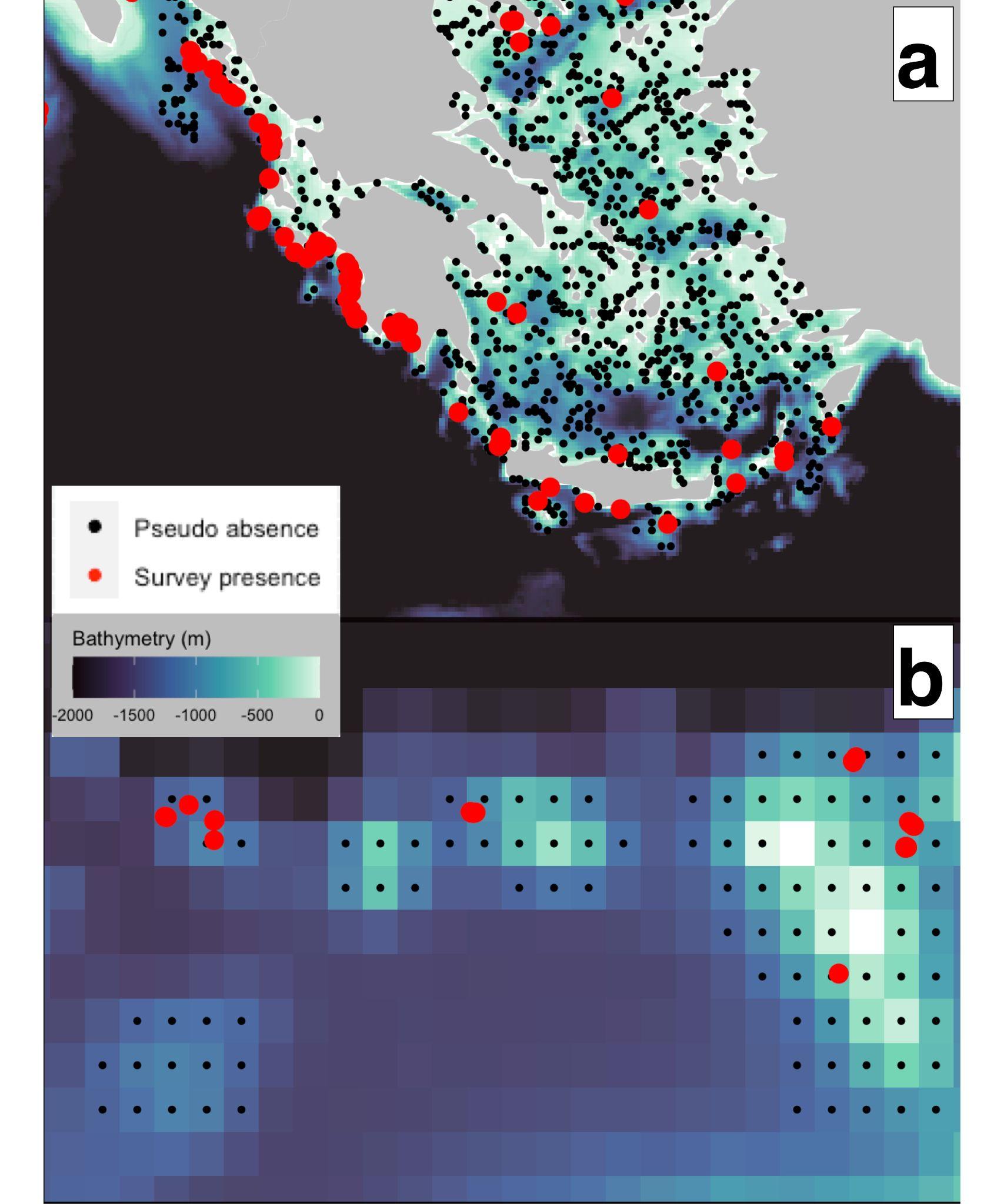


Figure S6: Pseudo absences added to the dataset in (a) : Northern-Ionian/aegean Sea and (b) aeolian islands Made using ggplot2 v3.4.4 https://cran.r-project.org/web/packages/ggplot2/index.html within the R environnement (R Core Team, 2023).

**Selecting spatial blocks for cross validation**

In order to partition the data between training and testing, we used the BlockCV R package, which requires setting a minimum distance for spatial blocks of the data, to confidently assume spatial independence and remove spatial autocorrelation.

This block size was chosen by running the cross validation procedure detailed in the material and methods section with different block size, and each time the mean AUC of the 5fold cross-validation is measured, to monitor the relationship between model performance and block size. The minimum block size was determined graphically as the inflexion point in the curve where model performance stops decreasing and decorrelate itself with block size.


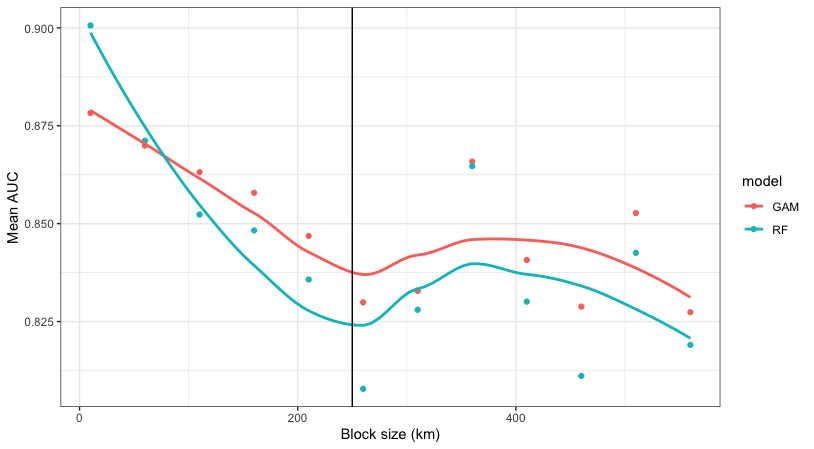


Figure S7: Mean model Area under the ROC curve in relation with cross validation block size. the vertical black line represent the retained chosen block size at 250 km. Made using ggplot2 v3.4.4 https://cran.r-project.org/web/packages/ggplot2/index.html within the R environnement (R Core Team, 2023).


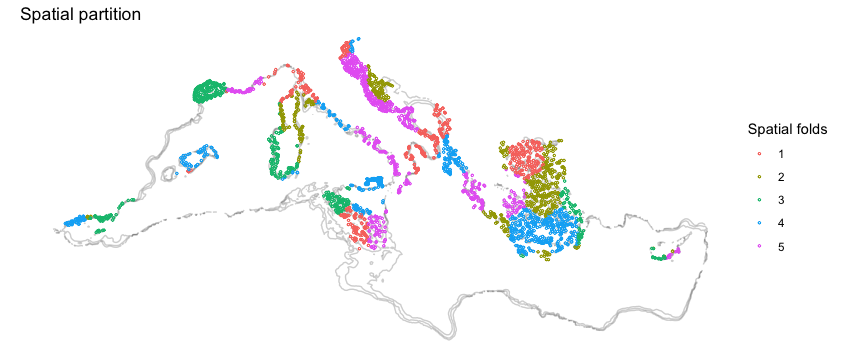


Figure S8: Example of fold assignation on the aggregated dataset for cross-validation using 250km block size. Made using ggplot2 v3.4.4 https://cran.r-project.org/web/packages/ggplot2/index.html within the R environnement (R Core Team, 2023).

**Model performance details**

Table S4 : Model evaluation of the 300 constructed models

| Metric | AUC | TSS | Sensitivity | Specificity |
| --- | --- | --- | --- | --- |
| Mean | 0.83 | 0.58 | 0.84 | 0.74 |
| Standard deviation | 0.03 | 0.05 | 0.06 | 0.06 |
| 1st quartile | 0.82 | 0.55 | 0.8 | 0.70 |
| median | 0.84 | 0.58 | 0.85 | 0.74 |
| 3st quartile | 0.86 | 0.62 | 0.88 | 0.79 |
| Minimum | 0.75 | 0.44 | 0.67 | 0.55 |
| Maximum | 0.89 | 0.71 | 0.97 | 0.87 |

Table S5 : Model evaluation of the 225 models retained in the ensemble modeling

| Metric | AUC | TSS | Sensitivity | Specificity |
| --- | --- | --- | --- | --- |
| Mean | 0.84 | 0.6 | 0.84 | 0.75 |
| standard deviation | 0.02 | 0.04 | 0.06 | 0.06 |
| 1st quartile | 0.83 | 0.57 | 0.81 | 0.72 |
| median | 0.84 | 0.60 | 0.86 | 0.76 |
| 3st quartile | 0.86 | 0.63 | 0.89 | 0.8 |
| Minimum | 0.82 | 0.49 | 0.67 | 0.58 |
| Maximum | 0.89 | 0.71 | 0.97 | 0.87 |


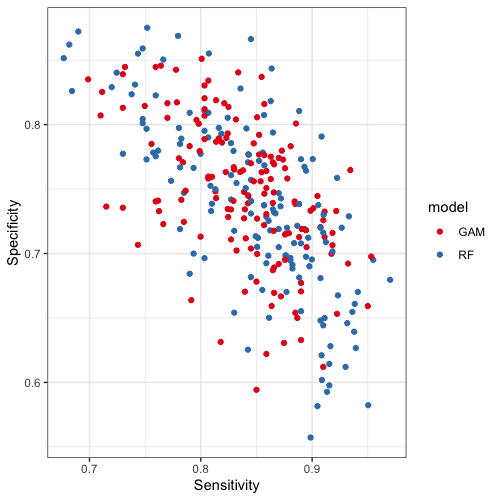

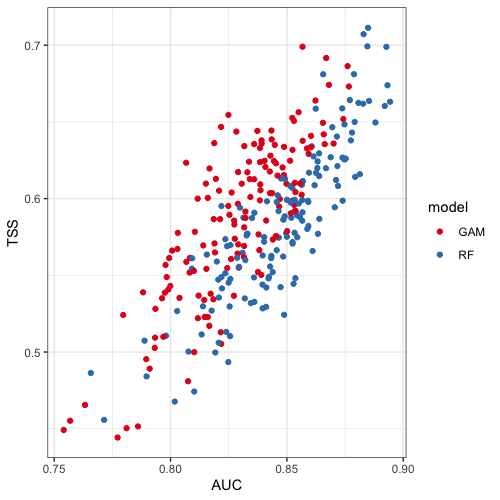


Figure S9: Performance metrics of all models constructed. Made using ggplot2 v3.4.4 https://cran.r-project.org/web/packages/ggplot2/index.html within the R environnement (R Core Team, 2023).
